# Catastrophic Health Expenditure and Rural Household Impoverishment in China: What Role Does the New Cooperative Health Insurance Scheme Play?

**DOI:** 10.1371/journal.pone.0093253

**Published:** 2014-04-08

**Authors:** Ye Li, Qunhong Wu, Chaojie Liu, Zheng Kang, Xin Xie, Hui Yin, Mingli Jiao, Guoxiang Liu, Yanhua Hao, Ning Ning

**Affiliations:** 1 Department of Social Medicine, School of Health Management, Harbin Medical University, Harbin, Heilongjiang, People's Republic of China; 2 School of Public Health, La Trobe University, Bundoora, Australia; Old Dominion University, United States of America

## Abstract

**Objective:**

To determine whether the New Cooperative Medical Insurance Scheme (NCMS) is associated with decreased levels of catastrophic health expenditure and reduced impoverishment due to medical expenses in rural households of China.

**Methods:**

An analysis of a national representative sample of 38,945 rural households (129,635 people) from the 2008 National Health Service Survey was performed. Logistic regression models used binary indicator of catastrophic health expenditure as dependent variable, with household consumption, demographic characteristics, health insurance schemes, and chronic illness as independent variables.

**Results:**

Higher percentage of households experiencing catastrophic health expenditure and medical impoverishment correlates to increased health care need. While the higher socio-economic status households had similar levels of catastrophic health expenditure as compared with the lowest. Households covered by the NCMS had similar levels of catastrophic health expenditure and medical impoverishment as those without health insurance.

**Conclusion:**

Despite over 95% of coverage, the NCMS has failed to prevent catastrophic health expenditure and medical impoverishment. An upgrade of benefit packages is needed, and effective cost control mechanisms on the provider side needs to be considered.

## Introduction

In 2012 China's population numbered 1.37 billion. Despite rapid urbanization over the past decade, half of the total population resides in non-urban areas. A large disparity in wealth and health status exists between urban and rural populations, resulting from a dualistic economic structure compounded by uneven social development and distance to amenities [1]. Compared to their urban counterparts, rural residents have lower levels of remuneration, relatively lower social security welfare entitlements and less access to services. This is a paradox: the government is determined to achieve social equality in a developing society where progress has unintended consequences.

The lower socio-economic status and paucity of social security for rural households increases their likelihood of falling into a downward cycle of “poverty and ill health”. International evidence has shown that people of lower economic status are more likely to suffer from serious illness and may become impoverished due to the medical costs [2].

The development of the New Rural Cooperative Medical Scheme (NCMS) represents the determination of the Chinese Government to interrupt this negative cycle of “poverty and ill health”. The NCMS, a community based financing scheme, aims to assist rural residents meet their medical costs. It is the successor to the Cooperative Medical Scheme (CMS). The CMS commenced in the 1950s and was predominantly financed and managed by peoples' communes. Concordant with the ideology of collective economies, CMS contributory members enjoyed primary health care without personal costs, provided predominantly by the “barefoot doctors”, a non-professionally trained stream of village health workers. At the time, the Chinese CMS model was widely recognized as one of the best in the world by international organizations, including the World Health Organization (WHO) [3]. By mid-1970s, the CMS extended to over 90% of the rural population [4]. Despite the modest dimensions of this service, population health outcomes steadily improved. At a cost of only 1% of total health resources of the world, China maintained an excellent record of health for a population that accounted for 22% of the world population [5]. When the collective commune economy began to disintegrate as a result of market-oriented economic reforms introduced in late 1970s, the CMS fell apart. Local governments were no longer keen to maintain CMS. CMS membership coverage dropped from 90% in 1978 to 7% in 1999 [6], [7]. This virtual collapse of the CMS resulted in an inability to remunerate barefoot doctors, who naturally lost any incentive to continue with health provision. Consequently, rural residents increasingly sought medical attention when needed from other more expensive facilities, necessitating consumer out-of-pocket (OOP) payments. Access to health care services became predicated on ability to pay, leading in some cases to catastrophic health expenditure (CHE) and impoverishment from medical expenses [8], [9].

By definition, CHE is where the burden of OOP health expenditure has reached a certain level that a household must forego expenditure on the needs of basic living in order to meet the medical expenses for one or more of the household members. A household is considered to be impoverished by medical expenses (medical impoverishment) when its available income falls below the poverty line [10].

In 2002, the Chinese Government decided to reform the CMS as the NCMS as a contributory strategy to alleviate medical impoverishment. The NCMS differs from the traditional CMS in respect of co-financing arrangements and insurance-like management of funds. Both local and regional government and contributory members contribute financially, which is supplemented by subsidies from the central government. Along with the rapid economic growth, the NCMS contribution fees rose from an average of 10 Yuan per capita in 2003 to 50 Yuan in 2004 and almost 100 Yuan in 2008 [11]–[13]. NCMS funds are managed at the county level. Consequently each fund may have its own benefit policy in terms of levels of deductibles, co-payments, and limits on reimbursement or payments to contributory members [14]. Due to variances in regional economic development, the overall available pooled funding varies, as do the benefits available to contributory members. Those residing in more affluent regions enjoy higher benefits, as their local governments are able to contribute more to the NCMS funds.

Membership of a NCMS is voluntary. But with strong governmental commitment, the scheme expanded from 333 participating counties in 2004 to 2728 counties in 2008, representing an increase of coverage from 80 million (75.2%) to 815 million (91.5%) rural residents [15], [16]. This in turn has fuelled a significant increased consumption of health services due to a previously latent unmet demand. From 2003 to 2008, the inpatient hospital admission rate for rural residents almost doubled, increasing from 3.38% to 6.75% [16]. The rapid expansion of NCMS coverage has been accompanied by soaring medical expenditure. From 2004 to 2010, per capita average annual health expenditure of rural residents rose by 54.7%, from 301.6 to 666.3 Yuan [12], [17], outpacing the rise of average household incomes of 50.2% from 4039.6 to 8119.5 Yuan [18], [19].

To date, effective cost control mechanisms on the provider side has been absent in China. Health services are remunerated by fee-for-service. Consumers are responsible for meeting expenses excluded from the benefit packages as determined by the NCMS policies, which may include specific services, deductibles, copayments and any charges exceeding the maximum compensatory payout. Given that consumer spending accounts for about 40% of total health expenditure in China, it remains unclear whether, as an economic strategy, the NCMS would be able to offer either protection or economic value for its contributory members, or of its intended strategic response towards the a reduction in CHE.

Although some researchers have attempted to evaluate the role of NCMS in addressing CHE [20], [21], conclusive results are as yet unavailable. There is a conspicuous paucity in the literature depicting the national picture of the NCMS as an instrument for the prevention of CHE and medical impoverishment. In this study, we selected a representative sample of national data to identify factors associated with CHE. We analyzed the factors associated with CHE occurrence in the context of the NCMS.

## Methods

### Data source and sampling method

Data used in this study were obtained from the fourth National Health Service Survey (NHSS, 2008). The NHSS is a regular sampling survey organized by the Central Government every five years, which features reliable quality control procedures. It was one of the most authoritative health survey data of China, this survey has been conducted every five year with its whole sampling design going through very rigid statistical examination and carried out through several round of experts consultation as well as under the technical guidance of statistical experts from WHO.A robust multi-stage and stratified random cluster sampling method was adopted to ensure a national representation of these surveys. The Fourth National Household Survey has required each province to survey the same numbers of units within each layer in order to secure the consistency on each layer. Systematic random sampling occurred at the following stages:

1. Municipalities (including both urban and rural geographic catchments) were categorized and ranked into five groups according to ten social-economic indicators such as geographic location, population size, economic, education and health status;
2. Some 94 municipalities were selected from the five groups;
3. Five townships/districts in each municipality were selected (470 townships/districts in total);
4. Two administrative villages/streets were selected from each sample township/district (940 sample villages/streets in total);
5. 60 households were selected from each sample village/district (56,400 households in total).

Staff from local medical organizations were recruited and trained to undertake face-to-face household interviews using a standardized questionnaire. The questionnaire collected demographic information of all household members and their health status (illness in previous two weeks and presence of chronic disease over the past six months), health service utilization (outpatient service utilization in previous two weeks and inpatient service utilization during the past year); as well as household income and food and health costs. Supervision of investigators ensured quality of data and 5% of sampled households were revisited to examine consistencies of data (>95% was achieved).

After the survey, the Health Statistical Center of the Ministry of Health of China (MOH) has made a systematic test and evaluation on the quality and representativeness of the survey data, Compared with the 2007 National Sampling Survey of Population Change, the representative of the original sample showed a good representation to the overall population, economy, education, and health status of the whole country. Myer's Index was 3.48, which showed no age bias existed. The Delta Dissimilarity Index was 0.04 and Gini Concentration Ratio was 0.016, which showed good consistency in the population distribution between the surveyed population and the general population [22].

### Ethics Statement

All the participants in this study obtained and signed the informed consents. And the Ethics Review Board of Harbin Medical University has approved this study.

### Statistical analysis

Only rural household data were analyzed for the purpose of this study. A small number of returned questionnaires with incomplete or anomalous data were excluded. This yielded a final sample of 38,945 households (129,635 people) for analyses.

Logistic regression analyses were performed, with the binary indicators of “percentage of households with CHE” as dependent variables. The WHO recommended method for calculating CHE and medical impoverishment was employed [10], which was based on the following expenditure variables:

1. Out-of-pocket health expenditure payments (OOP): payments made by a household for their health services without compensations from a third party;
2. Household consumption expenditure (EXP): payments and in-kind contributions made by a household for all goods and services, including the monetary value of home-made products;
3. Household subsistence expenditure (SE): household SE was calculated using food expenditure as a share of total household consumption expenditure. The weighted average food expenditure of a household, whose food expenditure as a share of household consumption expenditure fell between the 45^th^ and 55^th^ percentiles of the entire sample, was treated as the poverty line. The SE of each household was calculated as the poverty line multiplied by the standard household size of the household. Considering the economy scale of household consumption [10], (standard household size) = (actual household size)^0.56^;
4. Household's capacity to pay (CTP): non-subsistence spending of a household as a share of total household consumption expenditure.

CHE was defined as OOP payment for health care ≥40% of household CTP. Medical impoverishment indicates that a household becomes impoverished after paying for health services. When consumption expenditure is equal to or higher than SE, but is lower than SE net of OOP health payments, the household was considered impoverished [10].

In the analyses, we divided households into quintiles according to the household consumption expenditure. It is worth noting that the consumption quintile was ranked by equivalized per capita household consumption expenditure weighted with the standard household size instead of the actual household scale. Apart from the consumption quintile, independent variables entered into the regression analyses included: gender, education, employment and insurance status of the head of household, household size, age of the household members (whether the household having members aged above 60 years-old or below 5 years-old) and health of the household members(whether the household having at least one member with chronic disease, tuberculosis or admitted to hospital). According to the definition of WHO, we defined chronic disease including heart disease, hypertension, stroke, cancer, chronic respiratory diseases and diabetes which have long duration and generally slow progression. All of the analyses were performed using SAS (SAS Institute Inc., USA).

## Results

### Health service utilization and catastrophic health expenditure

In 2008, some 17% of respondents reported acute illness over the past two weeks preceding the survey. More than 14% of respondents reported presence of chronic disease over the past six months and 36.6% of households had one or more members with chronic disease. Among those people with acute illness, 30.7% did not seek medical care due to financial barriers; 29.0% chose self-treatment without a consultation from doctors and 16.3% did so because of concerns of medical expenses. The average cost for treating acute illness was 270.8 Yuan per patient and 67.2% of patients paid for those expenses out-of-pocket.

The hospital admission rate of respondents (during the past year) was 6.7%, which involved 16.6% of households. More than a quarter (27.5%) of patients refused admissions to hospitals mainly (71.3%) because of concerns of financial difficulties. The average annual cost for inpatient services was 2990 Yuan per patient, among which only 1106 Yuan were compensated by the insurance schemes.

According to the recommendations of the WHO, we converted the expenditure data into monthly based (Table 1). The average monthly OOP payment for health care increased with the household consumption expenditure, ranging from 41.6 Yuan for the lowest socio-economic status households to 321.3 Yuan for the highest. The average capacity to pay showed a similar trend, increasing from 190.3 Yuan for the lowest socio-economic status households to 1594.0 Yuan for the highest. Despite a higher OOP spending on health from the highest socio-economic status households, its share in capacity to pay was actually lower than that of the poorer households. Interestingly, the highest socio-economic status households had the highest share of OOP payment for health care in household consumption expenditure, consistent with its highest usage of hospital services. Both lowest (16.4%) and the highest (14.7%) socio economic status households were more likely to be affected by CHE than those in the middle range (Table 1).

**Table 1:** Health service utilization and expenditure of rural households, China 2008.

Use of and spending on health care	Household consumption per capita quintile					Total
	Lowest	2	3	4	Highest	
Usage of hospital services (%)	10.8	13.6	16.3	17.5	24.3	16.5
Average OOP payment (Yuan)	41.6	70.4	95.3	128.2	321.3	131.2
Average capacity to pay (Yuan)	190.3	334.2	472.4	706.9	1594.0	659.7
OOP share in household consumption expenditure (%)	13.2	12.2	12.0	11.8	14.1	12.7
OOP share in capacity to pay (%)	22.9	21.1	20.0	17.9	18.0	19.9
Household with CHE (%)	16.4	14.8	13.8	12.5	14.7	14.4

### Factors associated with catastrophic health expenditure

The households with higher needs of health care, such as those having one or more members older than 60 years, with chronic disease, or being admitted to hospitals were 2.4 to 3.3 times more likely to experience CHE (*p*<0.01) (Table 2).

**Table 2:** Percentage of households with catastrophic health expenditure across household consumption quintiles in rural China, 2008.

Household characteristics	Household consumption per capita quintile					Total	*p* value (Comparison across consumption quintile)
	Lowest	2	3	4	Highest		
One or more members with chronic disease							
Yes	27.7	27.2	25.3	22.7	24.5	26.0 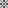	<0.01
No	10.2	8.4	7.6	6.5	7.9	8.1*	<0.01
One or more members older than 60 years							
Yes	23.7	22.3	21.1	18.2	21.2	21.9	<0.01
No	7.9	8.7	9.1	8.9	11.0	9.2*	<0.01
One or more members admitted to hospitals							
Yes	28.4	32.0	34.4	32.3	41.3	35.0	<0.01
No	15.7	12.3	10.0	8.2	6.3	10.7*	<0.01
Health insurance scheme of household head							
MIUE	13.0	12.5	10.5	8.5	11.0	10.8	*NS*
MIUR	19.0	10.0	8.7	6.3	2.0	6.4	<0.05
NCMS	16.5	15.0	14.0	12.7	15.4	14.9	<0.01
Others	-	8.3	17.6	23.5	11.5	13.6	*NS*
No insurance	15.4	12.4	12.7	10.8	11.6	13.0*	<0.05

Note: MIUE – Medical Insurance for Urban Employees; MIUR – Medical Insurance for Urban Residents; NCMS – New Cooperative Medical Scheme for Rural Residents. *NS – non statistically significant;*

* - *p*<0.01 compared with the households with different characteristics.

The association between household consumption expenditure and CHE showed a mixed picture. In general, the likelihood of a household experiencing CHE decreased with household consumption expenditure. However, the highest socio-economic group was less likely to deny admissions to hospitals and therefore was more likely to experience CHE when they had members admitted to hospitals compared with their lower socio-economic counterparts. The percentage of households with CHE increased from 28.4% for the poorest to 41.3% for the richest when one or more members of those household were admitted to hospitals.

Households headed by a person enrolled as a member of the NCMS had a similar (slightly higher) level of experience of CHE as those without insurance, indicating no protective effect. Conversely, the households headed by a member covered by the Medical Insurance for Urban Residents (MIUR) experienced a significant pro-rich effect on CHE, with households in the richest quintile having significantly lower instance of CHE (*P<0.05*) (Table 2).

When all of the above independent variables entered into the logistic regression model, all of them remained to be statistically significant (Table 3). The model proved that the lowest socio-economic quintile of households had a similar level of risk of CHE as their richest counterparts (p>0.50). Whereas, the households in the middle range of consumption expenditure experienced significantly less prevalence of CHE than the richest (OR = 0.69–0.79, p<0.001).

**Table 3:** Determinants of catastrophic health expenditure: a logistic regression model.

Household Characteristics	Sig.	OR	95% C.I.	
			Lower	Upper
One or more members with chronic disease (Yes vs. No)	<.0001	2.82	2.65	3.01
One or more members admitted to hospitals (Yes vs. No)	<.0001	4.80	4.46	5.16
One or more members with tuberculosis (Yes vs. No)	<.0001	1.68	1.33	2.13
One or more members older than 60 years (Yes vs. No)	<.0001	1.90	1.77	2.04
Number of household members (≥5 vs. ≤4)	<.0001	0.47	0.43	0.50
One or more members younger than 5 years (Yes vs. No)	0.004	0.82	0.72	0.94
**Household consumption per capita quintile**				
Lowest vs. Highest	0.605	0.97	0.86	1.09
Quintile 2 vs. Highest	0.112	0.91	0.80	1.02
Quintile 3 vs. Highest	0.000	0.79	0.70	0.90
Quintile 4 vs. Highest	0.000	0.69	0.61	0.79
**Demographic characteristics of household head**				
Gender (Male vs. Female)	0.021	0.91	0.83	0.99
Level of education				
None vs. University degree	0.009	14.77	1.94	112.36
Primary school vs. University degree	0.019	11.23	1.48	85.37
Junior high school vs. University degree	0.045	8.00	1.05	60.82
Senior high school vs. University degree	0.064	6.80	0.89	51.90
Vocational high school vs. University degree	0.083	6.16	0.79	48.14
Associate degree vs. University degree	0.126	5.13	0.63	41.74
Employment				
Employed vs. Unemployed	0.000	0.60	0.55	0.66
Retired vs. Unemployed	0.312	0.88	0.69	1.13
Student vs. Unemployed	0.124	0.53	0.24	1.19
**Health insurance scheme of household head**				
MIUE vs. None	0.000	0.57	0.42	0.77
MIUR vs. None	0.000	0.35	0.20	0.61
NCMS vs. None	0.745	1.02	0.89	1.17
Other types vs. None	0.270	0.69	0.35	1.34

Note: MIUE – Medical Insurance for Urban Employees; MIUR – Medical Insurance for Urban Residents; NCMS – New Cooperative Medical Scheme for Rural Residents.

The households with a head covered by the NCMS (in this situation, usually all household members would be covered by the NCMS) offered no protective effect on the occurrence of CHE (p>0.50). However, the households with a head covered by one of the two urban insurance schemes (those households were likely to have members enjoying a mixed schemes of insurance) were much better off in terms of the prevalence of CHE compared with those without insurance coverage (OR = 0.35–0.57, p<0.001). Households headed by a male, with a job attainment also had lower instances of CHE than their counterparts (p<0.05). Interestingly, larger households were also associated with lower instances of CHE. Nevertheless, higher health needs remain a major determinant of CHE. Those households with elderly or chronically ill members were 1.9 and 2.82 times more likely to experience CHE than their counterparts. Hospital admission increased the risk of CHE by 4.8 times (Table 3).

### Medical impoverishment distribution

After purchasing health services, 9.2% of households became impoverished. Similar to CHE, medical impoverishment was also associated with health care need (Table 4). The impoverishment rates of households having one or more members older than 60 years, with chronic disease, or being admitted to hospitals were about twice (1.6–2.2) as high as those of others (*p<0.01*). Significant regional disparities exist. Households located in the affluent eastern region were less likely to be impoverished from medical expenses than those located in the less affluent western region (7.3% vs. 10.8%, *p*<0.01), clearly indicating an inverse association between medical impoverishment and economic development.

**Table 4:** Percentage of households impoverished from medical expenses across regions in rural China, 2008.

Household characteristics	Region			Total	*p* value (comparison across regions)
	Eastern	Middle	Western		
One or more members with chronic disease					
Yes	11.1	14.2	14.8	13.3	<0.01
No	5.1	6.6	8.5	6.9*	<0.01
One or more members older than 60 years					
Yes	10.3	12.4	12.7	11.8	*NS*
No	4.8	7.1	9.3	7.3*	<0.01
One or more members admitted to hospitals					
Yes	15.8	15.9	18.1	16.8	<0.05
No	6.0	7.9	9.1	7.8*	<0.01
Health insurance scheme of household head					
MIUE	1.5	14.5	2.1	3.3	*NS*
MIUR	1.6	3.0	6.7	3.2	*NS*
NCMS	7.7	9.2	11.2	9.5	<0.01
Others	6.7	14.3	2.5	5.8	*NS*
No insurance	7.1	8.9	8.6	8.3*	*NS*
Total	7.3	9.3	10.8	9.2	<0.01

Note: MIUE – Medical Insurance for Urban Employees; MIUR – Medical Insurance for Urban Residents; NCMS – New Cooperative Medical Scheme for Rural Residents; *NS – non statistical significance;*

* - p<0.01 compared with the households with different characteristics.

Again, the households headed by a member covered by the NCMS had a similar (slightly higher) level of experience of medical impoverishment as those without insurance. Whereas, those households headed by a member of an urban insurance schemes had significantly lower levels of impoverishment than those without any insurance whatsoever (*p<0.01*).

## Discussion

### Prevalence of catastrophic health expenditure and medical impoverishment in rural China

The proportion of households associated with CHE and medical impoverishment in rural China were 14.4% and 9.2%, respectively. These rates are higher than those found in urban areas of China [23]. They are also higher compared with other middle and low-income countries [24], [25]. In a comparative study of CHE across 59 countries, only Brazil and Vietnam were found to have over 10% of CHE prevalence, close to the high proportion of CHE in China [26]. This is not surprising given the high level of OOP payments for health care. In 2008, OOP payments accounted for 40% of the total health expenditure in China [27]. The share of OOP payments in capacity to pay in rural China reached 22.9%; but in Turkey it was only 2.89% [28]. According to the WHO, universal access to health care would fail when OOP payments exceed 30% of the total health expenditure [29]. Empirical evidence showed that few households would experience CHE when OOP payments dropped down to lower than 15% of the total health expenditure [26].

### Factors associated with catastrophic health expenditure in rural China

OOP payments for health care are not the only determinant of CHE. The occurrence of CHE depends on the financial capacity of a household, health services accepted (or not accepted) by the household members and share of health expenses by the household members and the society [26], [30].

One of the surprising findings of this study is that CHE did not decline with the increase of household socio-economic status measured by consumption expenditure. The most affluent households experienced a similar level of CHE as their least affluent counterparts. It is commonly believed that strong financial capacity of a household would provide an effective buffer for preventing CHE [31]–[34]. This is true; however, when OOP payments for health care are high, poor households may choose not to seek medical care when needed instead of becoming impoverished from medical expenses. On the contrast, rich households have few financial concerns for access to medical care, but could end up with CHE. This study revealed that 30.7% of rural residents did not seek outpatient services when needed because of financial difficulties and 27.5% did not use the needed hospital services. Unmet health care needs and service avoidance are more prevalent in the lowest socio-economic status households than in the highest socio-economic status households [35], [36].

Health care services impose a heavy financial burden on rural households in China. Therefore, whether or not to seek health care usually involves a shared decision made by household members, in which the head of household plays a critical role. In this study, we found that households headed by a female, an unemployed person or a person having little education were more likely to experience CHE.

One of the most important functions of insurance is to prevent catastrophic payments of the insured. Unfortunately, the NCMS, an insurance scheme that covers over 90% of rural residents, has failed to fulfill this function. CHE are prevalent in the households insured with the NCMS, especially among those with high health need. This study showed that CHE occurred in 34.8% of households with one or more members admitted to hospitals; 25.7% of households with one or members having chronic disease; and 21.5% of households with elderly members. Those households had a corresponding impoverishment rate of 16.8%, 13.3% and 11.8% from medical expenses, respectively. However, it should not be ignored that the moral hazard is commonly observed in health insurance schemes of China. The induced demand for medical services, prevailing in the current medical services system of China, could lead to the increase in OOP for households, which could aggravate the severity of the CHE. Thus, the effect of the NCMS would be weakened more or less [4], [37], [38].

When insurance schemes have failed to prevent CHE, household members have to share the risk and the financial burden. This study revealed that larger households have stronger capacities to withstand and absorb CHE than the smaller ones. However, the ability of any single household to manage the risk of CHE is limited [39], [40]. Foregoing needed health care may help a household avoid CHE in the short term, but impose even greater risks for health and health expenditure in the future. The wealthiest households may not be able to escape from CHE either, especially when they have an elderly member, a member with chronic disease or tuberculosis, or a member that needs to be admitted to hospital.

### How to improve the ability of the NCMS to prevent catastrophic health expenditure and medical impoverishment?

The NCMS has been conceived as one of the most important strategies to reducing financial burdens of health care of rural households in China [41]. However, the high proportion of rural households that have experienced CHE and become impoverished is a clear indication of inadequate financial arrangements of the NCMS.

To begin with, the funds available in the NCMS are insufficient to meet the needs of rural residents. Although the average contribution per capita (including governmental subsidies) to the NCMS has risen to over 200 Yuan [12], [13], increased by 20 times over the past ten years, the total revenue generated falls short of need. By 2009, the average health expenditure per capita for rural residents had surpassed 562 Yuan [13]. The low financing level and lack of funds has inevitably led to low levels of compensatory benefits. Our study revealed that only 36.9% of hospital expenses for inpatient services were recompensed by the NCMS.

Second, shortcomings exist in the managerial arrangements of the NCMS. Unlike the urban insurance, such as the Medical Insurance Scheme for Urban Employees (MIUE) and the MIUR, in which funds are pooled into a municipal account, the NCMS funds have been managed at a lower (county/district) level. The poor ability of cost sharing due to limited numbers of contributory members further restricts the ability of the NCMS to compensate their contributory members.

Third, the NCMS cost control mechanisms have been demand-side measures. Health providers are paid through fee-for-service, which provides a strong incentive for them to maintain high volumes of services (some of which may be unnecessary). To control payout of the NCMS funds, the NCMS have to define deductibles, copayments, and maximum compensatory payout for inpatient services. For those patients who need treatments in secondary or tertiary hospitals (most inpatient services are actually delivered in those hospitals), compensations are further reduced [37]. In addition, many NCMS funds do not cover outpatient expenses, rehabilitation and long-term care for the elderly at all. This may explain why households having an elderly member or a member with chronic disease, as well as those with one or more members admitted to hospitals, are more likely to experience CHE and medical impoverishment.

In order to offer a better benefit package for NCMS contributory members, the financing level of the NCMS needs to be increased. The NCMS benefit package should align policies across the whole spectrum of health care services, including primary care, hospital care and long-term aged care. The integrated policies are also important for preventing cost shifting games.

Meanwhile, stronger attentions on provider-side measures need to be paid to control cost of health care [7]. The Ministry of Health is currently developing a case-mix funding system based on Diagnosis Related Groups (DRGs), with an intention to integrate the urban and rural health insurance schemes in the long run. International evidence has demonstrated that a one purchaser (third party) system along with case-mix funding encourages efficiency of health providers. Supply-side cost control measures may eventually bring better benefits to consumers [38]. Currently, oversupply of expensive medical services has been a serious issue of concern in China. People have placed a very high expectation on the government to rectify those perverse incentives through better regulation and financial incentives [29].

For now, however, transitional strategies must be considered. One of the biggest risks of CHE is “admissions to hospitals”. Our study revealed that households with one or more members admitted to hospitals were 4.8 times more likely to experience CHE than those without hospitalized members. Given this, compensation for inpatient services needs an urgent upgrade. According to the international benchmark, at least 70–80% hospital expenses should be paid by the insurance [7], [29]. The NCMS alone may not be strong enough to achieve this goal. Consequently, the medical financial assistance scheme (MFA), a scheme designed to offer additional support to the poor households for their CHE, should play an important role. Unfortunately, the current MFA operates independently from the NCMS and has been restricted to a limited number of households with limited payout [35].

## Conclusion

Financial protection in health can serve as a safety net for households in the face of economic shock [34]. Undoubtedly, the Chinese government is determined to offer its citizens such protections. The rapid expansion of the NCMS to vast rural areas and over 90% enrolments of rural residents in the NCMS have symbolized a great progress towards the goal.

However, in terms of the benefits rural people have enjoyed from the NCMS, China still has a long way to go. CHE and medical impoverishment for rural Chinese people remain unproportionally high. It is too early to claim that China has achieved universal coverage of health care as is defined by the WHO [31].

The NCMS has failed to prevent CHE for rural households. The limited scope of benefit and low compensation for hospital services deserve greater attention from the government. The Chinese government has committed to increasing funding for the NCMS; however, the increased funds have to be accompanied by effective cost control measures from the provider side. Moreover, better coordination and alignment of policies are needed. It is desirable to integrate the NCMS and MFA.

There are limitations in this study. As a cross-sectional study, it is not able to reveal any reduction of CHE and medical impoverishment (if exists) as a result of the NCMS. However, this study has proved that CHE is probably a better indicator than coverage for monitoring and evaluating the achievements of the NCMS. The occurrence of CHE involves multiple actors and very complicated mechanisms. During the period of making study design and statistical plan, we only focus on analyzing those micro household level influencing factors of CHE such as household demographic characteristics, income, family members' health need and utilization factors etc. while failing to take into consideration of macro social economic factors such as regional geographical differences into our study. Besides, the household impoverishment is one of the possible consequence CHE while coexisting many other risks factors, so if we would analyze comprehensively the sequential outcome of CHE and household impoverishment, it would add more valuable results to this studies, but due to limitation of accessing to those data again, we failed to improve it further. Meanwhile, we didn't take survey weights into consideration of logistic regression model, the odds ratio of which might be smaller than that of considering weight into it [42]. Base on those limitations above, future studies in related areas that taking those issues into considerations are urgently needed.
